# Treatment decision-making and treatment experiences in men with metastatic castration-resistant prostate cancer

**DOI:** 10.2340/1651-226X.2025.42748

**Published:** 2025-03-24

**Authors:** Sandra Doveson, Per Fransson, Lena Axelsson, Agneta Wennman-Larsen

**Affiliations:** a Department of Nursing Science, Sophiahemmet University, Stockholm, Sweden; b Department of Health Care Science, Marie Cederschiöld University, Stockholm, Sweden; c Department of Nursing, Umeå University, Umeå, Sweden; d Division of Insurance Medicine, Department of Clinical Neuroscience, Karolinska Institutet, Stockholm, Sweden

**Keywords:** Castration-resistant, communication, decision-making, prostate neoplasm, therapeutics

## Abstract

**Background and purpose:**

For the most advanced stage of metastatic castration-resistant prostate cancer (mCRPC), several life-prolonging treatments have become available over the past decade. Treatment decision-making (TDM) and experiences in this phase are yet to be studied. Hence, this study aimed to describe men’s satisfaction with TDM and treatment experiences during the first 12 months of a life-prolonging treatment of mCRPC.

**Patients and methods:**

This prospective study included 104 men with mCRPC who started and remained on the same life-prolonging treatment for 12 months. They received a questionnaire on TDM, treatment experiences, and well-being every 3 months. Correlation analyses explored associations between satisfaction with TDM at baseline and treatment experiences and well-being over time.

**Results and interpretation:**

The participants (median age: 77 years) generally reported high satisfaction with physician- and nurse communication and confidence/trust at baseline (>55% reported the highest satisfaction in all questions), but lower satisfaction with communication regarding how the treatments could affect them – up to 40% reported not having talked about that at all. Treatment experiences and physical- and emotional well-being remained stable over time. Associations were found between satisfaction with TDM at baseline and how they rated the treatment as a whole at six months, and well-being at six and 12 months. In mCRPC, men’s TDM preferences need to be explored, and shared decision-making needs to be facilitated when considering treatment. Furthermore, clinicians need to discuss how the treatment might affect patients’ everyday lives when discussing life-prolonging treatments with them.

## Introduction

About 1.5 million men around the world are diagnosed with prostate cancer (PC) yearly, making it the fourth most common cancer globally [1]. At its metastatic stage, symptoms, functioning, and quality of life (QoL) gradually worsen over time compared to men with non-metastatic PC [2]. For metastatic castration-resistant prostate cancer (mCRPC), the first real possibility for life-prolonging treatments came in 2004 with the use of Docetaxel chemotherapy to extend survival [3]. Over the first years following this breakthrough, chemotherapy served as the main treatment option with life-prolonging intent [4]. With the approval of life-prolonging hormone therapies [5, 6], the treatment landscape for mCRPC has swiftly evolved and expanded, and today, there are several treatment options available as both first- and consecutive life-prolonging treatment lines [7]. At this advanced incurable disease stage, decision-making regarding life-prolonging treatment means deciding whether to proceed with a treatment or not and, if so, deciding which treatment(s) may be appropriate. It involves a careful trade-off between the desired life-prolonging effects and intrusive side effects [8].

Treatment decision-making (TDM) among men with localized PC has been studied extensively [9–12], whereas decision-making in metastatic disease is far less researched. Previous research has found that many patients with cancer prefer a shared decision-making approach [13, 14], meaning the decision is made collaboratively with their physician [15], not least patients with advanced cancer [16]. In contrast, patients with localized PC might instead be involved in their TDM to a greater extent than they may have wished [13]. Men with PC who relapse after having undergone curatively intended treatment experience TDM differently when compared to their initial PC treatment. They experience more concerns about the illness’ treatability and rely more heavily on their physician’s treatment recommendation than they did before [17]. Furthermore, previous research also shows that a discrepancy between the desired and actual decision-making role is associated with poorer health-related QoL in patients with cancer [18]. Men’s TDM has also been studied in relation to later decisional regret in the localized phase of the PC disease [19–21]. Decisional regret has been found to be associated with unmet expectations about the treatment of localized PC, implying a discrepancy between what the participants expected and then experienced regarding treatment side effects [20]. Studies on decisional regret among men with mCRPC who undergo life-prolonging treatment are lacking.

In summary, although there is a lot of research and knowledge available on men’s TDM at the early stages of PC, life-prolonging treatment of mCRPC is a relatively young field, and TDM has yet to be studied to the same extent in this context. TDM at this disease stage is further complicated since mCRPC is incurable, which means that TDM involves a trade-off between possible benefits of life-prolonging treatments, life expectancy, and potential impact on everyday life and QoL. Consequently, it becomes important to consider potential associations between TDM experiences and how life-prolonging treatment is perceived over time. To our knowledge, this has not been studied before. Hence, this study aimed to describe men’s satisfaction with TDM and treatment experiences during the first 12 months of a life-prolonging treatment of mCRPC.

## Patients and methods

### Design

The present study is a prospective, multicenter cohort study. It uses real-world data from an oncology setting and complies with the ESMO Guidance for reporting oncology real-world evidence (Supplementary Material).

### Setting and participants

One hundred and fifty-four men who had been diagnosed with mCRPC were included consecutively at four oncology clinics in three cities from different regions in Sweden between 2015 and 2022 as they were about to start their first life-prolonging treatment of mCRPC. Inclusion criteria were:

- men starting all types of disease-directed treatments with life-prolonging intent
- men who were able to understand and express themselves in Swedish.

One hundred and seventy-six eligible men were informed and asked to participate by a research nurse/study coordinator at the study sites, whereafter a written informed consent was obtained from all who wished to participate.

### Data collection and measures

**Figure 1:**
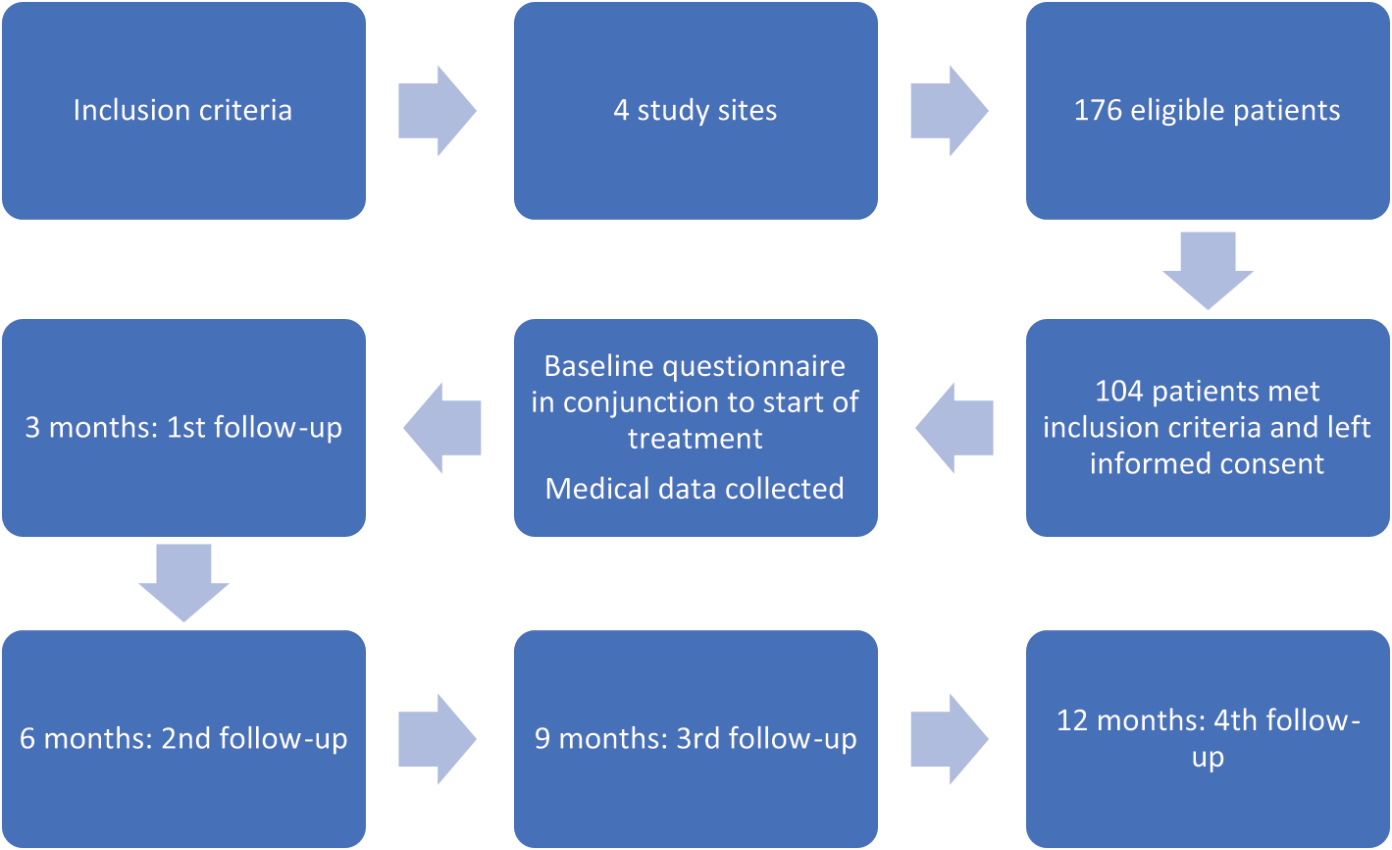
Flowchart over study process – recruitment of participants and data collection.

Data on TDM, treatment experiences, and well-being were obtained via paper questionnaires delivered by post. The participants received the first questionnaire upon inclusion in the study. They then received questionnaires every three months over the course of 12 months following inclusion (Figure 1). Medical data (age, year of diagnosis, time from diagnosis to inclusion in the study, Gleason score at diagnosis, site of metastases, blood serum levels of prostate-specific antigen (PSA) at diagnosis, and first life-prolonging treatment) were obtained from medical records upon inclusion in the study.

Physical and emotional well-being was measured at baseline and at all follow-ups using the validated instrument Functional Assessment of Cancer Therapy - General (FACT-G) [22, 23]. The instrument consists of 27 items with statements about well-being. It has a 5-point Likert-type response scale containing the response alternatives ‘Not at all’, ‘a little bit’, ‘somewhat’, ‘quite a bit’, and ‘very much’. The instrument is divided into four subscales: physical, emotional, functional, and social well-being [22]. Negatively phrased question scores are reversed, and scores for each item within a subscale are summed up to a total subscale score, with a higher score indicating better well-being. For this study, the subscales physical and emotional well-being were used.

Satisfaction with TDM and treatment experiences was measured using a study-specific instrument, since no instruments covering TDM and treatment experiences were available in Swedish at the time for planning and executing the study. The instrument was inspired by preexisting instruments available in English at the time [24, 25] and consists of two sections: one with questions regarding the participants’ satisfaction with TDM and one with questions regarding the participants’ treatment experiences. The section about satisfaction with TDM is present in the baseline questionnaire and consists of 27 questions on TDM, communication, confidence, and trust. The questions cover interactions with both physicians and nurses and concern, for example, the participants’ satisfaction with the availability and sense of commitment from physicians and nurses; how treatment options, benefits, and risks had been explained; and the level of trust and confidence they felt in the physician. The questions also cover their satisfaction with the dialogue about how the treatment could affect them as well as satisfaction with whether their needs were understood, and if they were able to ask questions and bring forth what they felt was important in their situation. The instrument uses a four-step Likert-type response scale that ranges from ‘No, not at all’ to ‘Yes, as much as I wanted to’. The TDM items are divided into subscales in the analysis, and this study utilized the following subscales: satisfaction with physician communication, satisfaction with treatment staff communication, satisfaction with nurse communication, and satisfaction with confidence and trust. The score of each item within a subscale was summed, and the sum was then multiplied with the number of questions in the subscale. To obtain the total subscale score, the product was divided by the number of questions that had been answered in the subscale. Higher subscale scores indicated higher satisfaction. The instrument section about treatment experiences is present in all subsequent follow-up questionnaires and comprises eight questions. It uses a Likert-type response scale with 2–4 steps and responses ranging from ‘A lot worse/No, not at all/No/Bad’ to ‘A lot better/Completely/Yes/Excellent’. Four questions from this section were selected for this study, namely, ‘Do you think you are receiving the treatment that is right for you?’, ‘Would you recommend this treatment to others with the same illness as you?’, ‘Would you choose this treatment again?’ and ‘As a whole, how would you rate this treatment?’. The items in this section are treated as single items in analysis. The instrument was evaluated using think aloud interviews [26] and showed good face validity prior to the study [27].

### Data analysis

Descriptive statistics were calculated for medical and sociodemographic characteristics at baseline. Furthermore, descriptive statistics were calculated for the participants’ satisfaction with TDM subscales at baseline. Because data were unevenly distributed in most variables, medians (md) and interquartile ranges (IQRs) are presented. The development of treatment experiences, physical well-being, and emotional well-being (md, IQR) was calculated for baseline and all four follow-ups during the 12 months in the study. A correlation analysis (Spearman’s rank correlation, *r_s_*) was then performed to explore associations between satisfaction with TDM at baseline and treatment experiences and physical- and emotional well-being at both 6 and 12 months. A significance level of *p* < 0.05 was considered statistically significant in all analyses. All statistical analyses were performed using the software *IBM SPSS Statistics version 27* (IBM, Armonk, NY, USA).

### Ethics declaration

This study was approved by the Regional Ethical Review Board (now the Swedish Ethical Review Authority) in Stockholm, Sweden (Dnr 2014/341-31/2, Dnr 2016/851-32, and Dnr 2016/2230-32). All participants provided a written informed consent prior to participation in this study.

## Results

### Participant characteristics

One hundred and four participants started and remained on the same life-prolonging treatment over 12 months, hence constitute the sample for this study (Table 1). The men were mostly older [median age 76.5 (IQR 72.4–81.1)], and the majority (79.8%) were married and/or cohabitating with a partner. Most of them (91.3%) were born in Sweden, and their educational level ranged from 9-year compulsory school to university-level studies. Most had presented with intermediate to high-grade Gleason scores [median 7.0 (IQR 7.0–8.0)] at diagnosis. Upon inclusion in the study, the men’s median PSA value was 25.0 μg/L (IQR 11.0–59.8). The dominating sites for metastases were bone (66.3%) and lymph nodes (29.8%). As for the life-prolonging treatment, five different first-line treatments are represented in the sample even though the majority of the participants (79.8%) underwent hormone treatment (Abiraterone or Enzalutamide). Among those who underwent chemotherapy, the median length of treatment was 16 weeks (IQR 14.0–18.8) (Table 1).

**Table 1:** Participants’ medical and sociodemographic characteristics (*n* = 104).

Variable	Median	IQR
Age at inclusion (years)	76.5	72.4–81.1
Time from primary PC diagnosis to inclusion in study/baseline (months)	41.9	17.8–97.3
Gleason score at primary PC diagnosis Missing *n* = 5	7.0	7.0–8.0
PSA at inclusion in the study (μg/L)	25.0	11.0–59.8

	n	%

**Marital status**		
Single	10	9.6
Widowed	7	6.7
Married/in a relationship/cohabitating with partner	83	79.8
Other	1	1.0
Missing	3	2.9
**Place of birth**		
Sweden	95	91.3
Other Nordic country	3	2.9
Country outside of Europe	3	2.9
Missing	3	2.9
**Educational level**		
9-year compulsory school	42	40.4
High school	34	32.7
University	24	23.1
Missing	4	3.8
**Site of metastases**		
Bone	69	66.3
Lung	3	2.9
Lymph nodes	31	29.8
Other	1	1.0
**First life-prolonging treatment**		
Docetaxel	17	16.3
Cabazitaxel	2	1.9
Abiraterone	18	17.3
Enzalutamide	65	62.5
Radium-223	2	1.9

	Median	IQR

Length of treatment (weeks) (Docetaxel + Cabazitaxel)	16	14.0–18.8

PC: prostate cancer; PSA: prostate-specific antigen; IQR: interquartile range.

### Levels of satisfaction with TDM at baseline

**Figure 2:**
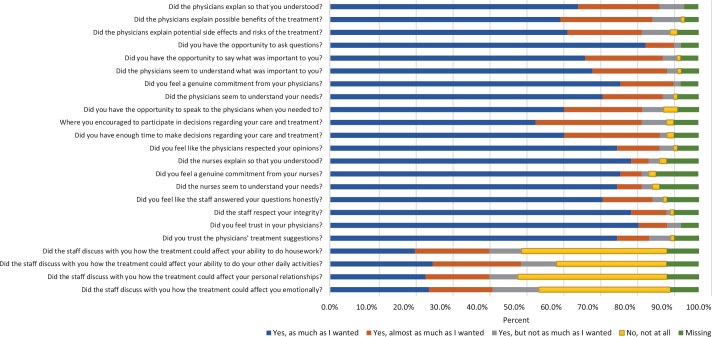
Distribution of responses to the satisfaction with treatment decision-making questions at baseline.

The participants generally reported high levels of satisfaction with the physician communication [median 34.0 (IQR 30.0–36.0)] (Table 2). Regarding physician communication, over 70% of the participants reported the highest level of satisfaction (‘yes, as much as I wanted’) in five questions, 60–70% of the participants reported the highest level of satisfaction in five questions, and for one question, 56% reported the highest level of satisfaction in whether they had been encouraged to participate in decisions regarding their care and treatment (Figure 2). The participants also reported high levels of satisfaction with the nurse communication [median 9.0 (IQR 9.0–9.0)] (Table 2), with over 75% of the participants answering ‘yes, as much as I wanted’ to all three questions (Figure 2). Furthermore, confidence and trust also reached a high score at baseline [median 12.0 (IQR 11.0–12.0)], indicating a high level of satisfaction (Table 2). In all four questions about confidence and trust, over 70% of the participants reported the highest level of satisfaction (‘yes as much as I wanted to’) (Figure 2). The participants reported lower scores on the treatment staff communication [median 5.0 (IQR 1.0–10.0)] dimension (Table 2). Their answers were also more evenly distributed over the response scale in this dimension (Figure 2). To the question if the treatment staff had discussed how the treatment could affect their ability to do housework, 39% responded ‘no, not at all’. About a third (30%) also responded ‘no, not at all’ to whether the staff had discussed how the treatment could affect their ability to do other daily activities. The response ‘no, not at all’ was also the most frequently reported to the questions regarding whether the staff had discussed how the treatment could affect personal relationships and affect them emotionally [40 and 36%, respectively].

**Table 2:** Satisfaction with treatment decision-making and development of treatment experiences and well-being from baseline, over approximately 12 months, to the fourth follow-up.

Variable	Baseline	1st follow-up	2nd follow-up	3rd follow-up	4th follow-up
Physician communication^a^					
Median (IQR)	34.0 (30.0–36.0)	-	-	-	-
*N*	99				
Treatment staff communication^b^					
Median (IQR)	5.0 (1.0–10.0)	-	-	-	-
*N*	95				
Nurse communication^c^					-
Median (IQR)	9.0 (9.0–9.0)	-	-	-	-
*N*	94				
Confidence and trust^d^					-
Median (IQR)	12.0 (11.0–12.0)	-	-	-	-
*N*	97				
‘Do you think you are receiving the treatment that is right for you?^e^	-				
Median (IQR)		3.0 (2.0–3.0)	3.0 (2.0–3.0)	3.0 (2.0–3.0)	3.0 (2.0–3.0)
*N*		65	62	61	43
‘Would you recommend this treatment to others with the same illness as you?^f^	-				
Median (IQR)		2.0 (2.0–2.0)	2.0 (2.0–2.0)	2.0 (2.0–2.0)	2.0 (2.0–2.0)
*N*		65	61	61	43
‘Would you choose this treatment again?^f^	-				
Median (IQR)		2.0 (2.0–2.0)	2.0 (2.0–2.0)	2.0 (2.0–2.0)	2.0 (2.0–2.0)
*N*		64	62	61	43
‘As a whole, how would you rate this treatment?^g^	-				
Median (IQR)		3.0 (2.0–4.0)	3.0 (2.0–4.0)	3.0 (2.0–4.0)	3.0 (2.0–4.0)
*N*		65	62	62	43
Physical well-being^h^					
Median (IQR)	23.0 (17.4–27.0)	22.0 (17.0–25.0)	23.0 (20.0–26.0)	24.0 (20.0–26.0)	23.0 (18.0–26.0)
*N*	102	90	82	74	51
Emotional well-being^i^					
Median (IQR)	19.0 (16.0–21.5)	20.0 (18.0–22.0)	20.0 (18.0–23.0)	20.00 (18.0–22.0)	20.00 (16.0–22.0)
*N*	101	89	83	73	51

IQR: interquartile range.

a Satisfaction with treatment decision-making – physician communication: higher values indicate higher satisfaction (range: 0–36).

b Satisfaction with treatment decision-making – treatment staff communication: higher values indicate higher satisfaction (range: 0–12).

c Satisfaction with treatment decision-making – nurse communication: higher values indicate higher satisfaction (range: 0–9).

d Satisfaction with treatment decision-making – confidence and trust: higher values indicate higher satisfaction (range: 0–12).

e Experiences of treatment: higher values indicate better treatment experience (range: 0–3).

f Experiences of treatment: higher values indicate better treatment experience (range: 0–2).

g Experiences of treatment: higher values indicate better treatment experience (range: 0–4).

h FACT-G physical well-being: higher values indicate better well-being (range: 0–28).

i FACT-G emotional well-being: higher values indicate better well-being (range: 0–24).

### Levels of satisfaction with the treatment over time

The men generally reported high scores (indicating a good treatment experience) [median 3.0 (IQR 2.0–3.0)] when asked if they thought they received the treatment that was right for them. The scores remained stable at that level throughout the 12 months. Similarly, they also reported high scores on whether they would recommend this treatment to others with the same illness [median 2.0 (IQR 2.0–2.0) across all follow-ups], and if they would choose the same treatment again [(median 2.0, IQR 2.0–2.0)]. The reported scores of the last question, how the men would rate the treatment as a whole, also remained stable over time from 3 to 12 months [median 3.0 (IQR 2.0–4.0)] (Table 2).

### Associations between satisfaction with TDM, treatment experiences, and well-being

Statistically significant associations were found between satisfaction with TDM at baseline and aspects of treatment experiences at 6 months (Table 3). Satisfaction with physician communication was associated with how the participants rated the treatment as a whole at 6 months [*r_s_*: 0.304, *p* = 0.019]. Associations were also found between satisfaction with nurse communication and whether they thought they received the treatment that was right for them [*r_s_*: 0.308, *p* = 0.024]. Furthermore, satisfaction with physician communication was associated with physical and emotional well-being at 6 months [*r_s_*: 0.407, *p* < 0.001 and *r_s_*: 0.478, *p* < 0.001, respectively]. Similarly, satisfaction with nurse communication at baseline was found to be associated with physical and emotional well-being at 6 months [*r_s_*: 0.290, *p* = 0.012, respectively]. Satisfaction with treatment staff communication was associated with emotional well-being at 6 months [*r_s_*: 0.244, *p* = 0.034]. Satisfaction with confidence and trust was associated with emotional well-being at 6 months [*r_s_*: 0.262, *p* = 0.021].

**Table 3:** Correlations* between satisfaction with treatment decision-making at baseline and treatment experiences and physical- and emotional well-being after 6 and 12 months.

Variable			Satisfaction with physician communication	Satisfaction with treatment staff communication	Satisfaction with nurse communication	Satisfaction with confidence and trust
6 months	‘Do you think you are receiving the treatment that is right for you?’	Correlation coefficient *p*	0.176 0.183	-0.003 0.982	**0.308** **0.024**	0.036 0.791
	‘Would you recommend this treatment to others with the same illness as you?’	Correlation coefficient *p*	0.074 0.583	-0.061 0.660	0.187 0.181	-0.038 0.780
	‘Would you choose this treatment again?’	Correlation coefficient *p*	0.240 0.067	-0.018 0.893	0.145 0.294	0.043 0.749
	‘As a whole, how would you rate this treatment?’	Correlation coefficient *p*	**0.304** **0.019**	0.029 0.833	0.262 0.055	0.100 0.461
	Physical well-being	Correlation coefficient *p*	**0.407** **<0.001**	0.179 0.124	**0.290** **0.012**	0.200 0.082
	Emotional well-being	Correlation coefficient *p*	**0.478** **<0.001**	**0.244** **0.034**	**0.290** **0.012**	**0.262** **0.021**
12 months	‘Do you think you are receiving the treatment that is right for you?’	Correlation coefficient *p*	0.115 0.469	0.160 0.330	-0.021 0.898	0.131 0.421
	‘Would you recommend this treatment to others with the same illness as you?’	Correlation coefficient *p*	0.077 0.630	-0.056 0.733	-0.159 0.339	0.264 0.099
	‘Would you choose this treatment again?’	Correlation coefficient *p*	0.288 0.065	0.041 0.803	-0.159 0.339	**0.407** **0.009**
	‘As a whole, how would you rate this treatment?’	Correlation coefficient *p*	0.096 0.546	0.148 0.369	0.158 0.344	0.047 0.776
	Physical well-being	Correlation coefficient *p*	0.204 0.155	0.020 0.894	0.207 0.167	0.228 0.119
	Emotional well-being	Correlation coefficient *p*	**0.477** **<0.001**	0.047 0.756	0.076 0.616	0.113 0.443

* Tested with Spearman’s rank correlation, *r_s_*. Correlations that were statistically significant are highlighted in bold.

At 12 months, satisfaction with physician communication at baseline was found to be associated with emotional well-being [*r_s_*: 0.477, *p* < 0.001] (Table 3). An association was also found between satisfaction with confidence and trust at baseline and whether the treatment would be chosen again at 12 months [*r_s_*: 0.407, *p* = 0.009].

## Discussion

This prospective study describes men’s satisfaction with TDM and treatment experiences during the first 12 months of a life-prolonging treatment of mCRPC. The men generally reported high satisfaction with TDM at the start of life-prolonging treatment and generally reported a high level of satisfaction with their treatment experiences, which was also stable over the first 12 months. Moreover, there are associations between aspects of satisfaction with TDM at the start of life-prolonging treatment and treatment experiences and well-being at 6 and 12 months after the start of treatment.

The men in the present study reported a high level of satisfaction with the physician and nurse communication when starting life-prolonging treatment. Previous research on localized PC shows that physician communication as well as trust and confidence in one’s physician is immensely important for men when making decisions about their treatment [9, 28–30]. A positive relationship has been shown between shared decision-making experiences, the physician’s explanations, and treatment satisfaction among men with metastatic PC who undergo hormone therapy [31]. Even though a TDM experience that could be described as shared [32] seems to be associated with better treatment experiences [31], research also shows that both patients and healthcare professionals sometimes lack confidence in the patient’s ability to partake in decision-making in the context of advanced PC. A consequence may be both parties expecting the physician to take the lead in the TDM process [33], which might lead to patients not partaking in TDM in the way they would have wanted.

The men in the present study reported high scores (indicating a good treatment experience) on whether they felt they were receiving the right treatment, if they would choose it again, and if they would recommend it to someone else with the same illness. The high scores remained stable during the 12 months. Previous research has found associations between type of curative treatment chosen for localized PC and decisional regret, but there is no consensus about the direction of the associations. One study revealed that men who had chosen a more invasive primary treatment (radical prostatectomy or radiation therapy) reported decisional regret to a greater extent than those who chose to undergo active surveillance [19]. A more recent study showed the contrary – men who had chosen surgery or radiation therapy reported less decisional regret than those who chose other treatments [21]. Choosing between curatively intended treatments, where the treatment effects have proven to be equivalent, is different than trying to determine whether a life-prolonging treatment will be worth it or not in the end, as seen in previous research on men with mCRPC [8].

A strength of this study is the prospective design, with repeated follow-ups over a prolonged period of time. To counteract selection bias, we chose to include participants from four different oncology clinics. A sample of only men who remained on the same treatment for 12 months was included in this study, which revealed a rather small sample size in relation to the power calculations performed for the research project in order to detect clinically meaningful changes using the FACT-G [34]. This might be considered a limitation of the study. However, given the advanced disease stage of the participants in this study, the power calculation was made taking attrition due to death of participants over time into consideration. As a result, the sample for this study comes in just short of the lower limit (*n* = 120) of required participants to reach a statistical power of 80%. It is, however, possible that a larger sample size could have enabled us to detect additional associations between variables over time, and hence, the study results need to be generalized with caution. Another aspect that needs to be considered is the attrition that occurred over the 12 months. It was mostly due to the death, or declining condition, of the participants, which implies that the participants with the most severe illness, therefore possibly declining treatment experiences, were not represented to the same extent over time. Furthermore, it is not uncommon for men with mCRPC to undergo more than one type of life-prolonging treatment, and the generalizability of the results from this study to these patients must be considered with caution. A strength of this study is using a well-validated instrument (FACT-G) [22] to measure well-being. A limitation is, however, that the second instrument measuring satisfaction with TDM and treatment experiences is study-specific and has not undergone psychometric testing. The study-specific instrument was, however, found to have face validity when tested using think aloud interviews [26] prior to the study [27].

## Interpretation

Men with mCRPC generally reported high satisfaction with TDM when starting a life-prolonging treatment. However, more attention needs to be directed toward communication regarding how the upcoming treatment might affect them and their everyday lives. This is a prerequisite for men with mCRPC to be able to make an informed decision about treatment. An important part of TDM for involved clinicians is also facilitating a shared decision-making process that adapts to the patient’s preferences and wishes regarding how to make treatment decisions. Shared decision-making, therefore, also needs to entail exploring men’s TDM preferences specifically – which role in TDM do they want? Decisional aids may serve as important and useful tools in fostering shared decision-making in this context. The need to adhere to the men’s preferred role in TDM is further underlined by the associations between satisfaction with TDM and treatment experiences and well-being over time in men with mCRPC on life-prolonging treatment.

## Supplementary Material

Treatment decision-making and treatment experiences in men with metastatic castration-resistant prostate cancer

## Data Availability

The datasets generated and analyzed during the current study are not publicly available due to the General Data Protection Regulations and the Swedish Ethical Review Act. For data that support the findings of this study, please contact author Agneta Wennman-Larsen.
